# *Microdaccus* Schaum, 1864: a new species from Oman, taxonomic notes and an identification key to the species of the genus (Coleoptera, Carabidae, Lebiinae)

**DOI:** 10.3897/BDJ.14.e185048

**Published:** 2026-08-10

**Authors:** Thorsten Assmann, Ali Abduallah Al-Jahdhami, Swantje Grabener

**Affiliations:** 1 Leuphana University Lüneburg, Lüneburg, Germany Leuphana University Lüneburg Lüneburg Germany; 2 Ministry of Agriculture and Fisheries Wealth, Samad Ashan, Oman Ministry of Agriculture and Fisheries Wealth Samad Ashan Oman

**Keywords:** Arabian Peninsula, Hajar Mountains, *Microdaccus birgitae* sp. nov., *
Psammodromius
*

## Abstract

**Background:**

*Microdaccus* Schaum, 1864 and *Psammodromius* Peyerimhoff, 1927 are regarded as two closely-related taxa of Lebiinae, comprising a total of 11 species. The species known to date occur from south-eastern Europe and Egypt to Kazakhstan, Afghanistan and Iran.

**New information:**

*Microdaccus birgitae* sp. nov. is described from the Hajar Mountains, Sultanate of Oman. Characterised by its slender pronotum with lateral margin almost not widened to hind angles and strongly rounded elytra, this species extends the known distribution range of the genus to the southern Arabian Peninsula. We studied taxonomically most of the *Microdaccus* and *Psammodromius* species, including some type material, re-assessing characters and rank Psammodromius Peyerimhoff, 1927 stat. nov. as a subgenus of *Microdaccus* Schaum, 1857. An illustrated key enables the identification of the known *Microdaccus* species.

## Introduction

The lebiine ground beetle genus *Microdaccus* has been erected by Schaum in 1864. Nine species of this genus are known from southern Balkan Peninsula to Kazakhstan, Afghanistan and Iran (Kabak 2017a, Muilwijk et al. 2019, Anichtchenko 2025). The related genus *Psammodromius* was described by Peyerimhoff (1927), based on a species from Egypt. Mateu (1981) and Kabak (2016) included a further species in this genus, bringing the total number of known species in both genera to eleven.

## Materials and methods

The following abbreviations are used: TL, total body length from tip of mandibles to apex of elytra; HW, maximum head width; HW-E, maximum head width minus eyes (minimum width of frons); AL, antennal length from basal incision of 1^st^ antennomere to apex of 11^th^ antennomere; PW, maximum width of pronotum; PC, minimal width of constriction in front of pronotal hind angles (this character is difficult to recognise in *M. birgitae* sp. nov.); PB, distance between pronotal hind angles; PL, maximum pronotal length along the median line; EW, maximum width of elytra; EL, elytral length from apex of scutellum to apex of right elytron.

Before measurements, we checked at higher magnification that the measured distances of the given body parts are exactly horizontal. At a magnification at about 100x, we examined microsculpture. We used standard techniques for dissections and preserved the genitalia in a polyvinylpyrrolidone-containing mixture (Lompe 1989) on a label pinned beneath the specimens from which we removed them.

We prepared photographs with an Olympus E-330 digital camera in combination with a Leitz MZ 95 microscope and with a Leica SL-2 camera attached to the macro-objective Laowa Ultra Macro 2.5-5.0x. For stacking (up to about 100 layers), we used the software Picolay (Cyprionka 2025). Photographs from material in NMBS are made with the digital camera KEYENCE VHX-7000 4K.

The specimens were not cleaned with liquids such as water, ethanol or detergents prior to photographing, nor were they remounted (for aesthetic reasons). We hope that this approach will not accelerate the degradation of the DNA (Mandrioli 2008). Rather, our photographs represent the status quo of the specimens in the given collections.

The type specimen of the new species mentioned in the description is deposited in the working collection of the first author, which is part of the Zoological State Collection Munich (ZSM). In order to create an identification key for the *Microdaccus* species, we examined the available material from the Natural History Museum Basel (NMBS), Natural History Museum in London (NHMUK), Muséum national d'Histoire naturelle in Paris (NHMN), Steinhardt Museum of Natural History in Tel Aviv (SMNH), Natural History Museum in Vienna (NHMW), as well as from the collection of the first author (working collection Assmann, Asendorf (district of Harburg); CAA).

## Taxon treatments

### Microdaccus (Microdaccus) birgitae

Assmann, Al-Jahdhami & Grabener sp. nov.

7F6BC331-8454-5941-8D13-CD268E506C0B

FCC3469D-D526-47C5-BFE0-2B63D3F3B4FF

#### Materials

- **Type status:** Holotype. **Occurrence:** individualCount: 1; sex: female; lifeStage: adult; behavior: active; occurrenceStatus: present; occurrenceID: 7379BDFF-2FB6-542A-8BB3-FB7396D87A16; **Taxon:** scientificName: *Microdaccus birgitae* Assmann, Al-Jahdhami & Grabener, 2026; acceptedNameUsage: *Microdaccus birgitae*; parentNameUsage: Lebiinae; kingdom: Animalia; phylum: Arthropoda; class: Insecta; order: Coleoptera; family: Carabidae; genus: *Microdaccus*; subgenus: *Microdaccus*; specificEpithet: *birgitae*; taxonRank: species; scientificNameAuthorship: Assmann, Al-Jahdhami & Grabener, 2026; **Location:** continent: Asia; country: Oman; countryCode: OM; stateProvince: Batinah; county: Rustaq; locality: Balad-Sayt; verbatimElevation: 1050 m; verbatimLatitude: 23.193 N; verbatimLongitude: 57.388 E ; verbatimCoordinateSystem: decimal degrees; decimalLatitude: 23.193; decimalLongitude: 57.388; **Identification:** identifiedBy: Thorsten Assmann; Ali A Al-Jahdhami; Swantje Grabener; dateIdentified: 2025; **Event:** eventID: ThAsOman24; eventDate: 15/03/2024; year: 2024; month: 3; day: 15; habitat: semi-arid submontane open xeromorphic woodlands and semi-evergreen shrubland; **Record Level:** type: PhysicalObject; modified: 2026-01-08; language: en; rights: CC BY 4.0; rightsHolder: Thorsten Assmann, Ali Abdullah Al-Jahdhami, Swantje Grabener; accessRights: not-for-profit use only; bibliographicCitation: Assmann T, Al-Jahdhami AA, Grabener S (2016) *Microdaccus* Schaum, 1864: a new species from Oman, taxonomic notes, and an identification key to the species of the genus (Coleoptera, Carabidae, Lebiinae). Biodiversity Data Journal.; institutionID: SNSB-ZSM; institutionCode: Leuphana University Lueneburg; basisOfRecord: PreservedSpecimen

#### Description

**Type locality**. Sultanate of Oman, Western Hajar Mountains, close to Balad Sayt.

**Type material**. Holotype: Female: “Oman: South Batinah, Hajar Mts / Balad Sayt, 1050 m a.s.l. / N23°11.6’ E57°23.3’ / 15/16.III.2024 / leg. B. & Th. Assmann” [white label] “HOLOTYPE / *Microdaccus birgitae* / spec. nov. / det. Th. Assmann & / A.A. Al-Jahdhami, / S. Grabener, 2025” [red label] (CAA, ZSM).

**Figure 1.**
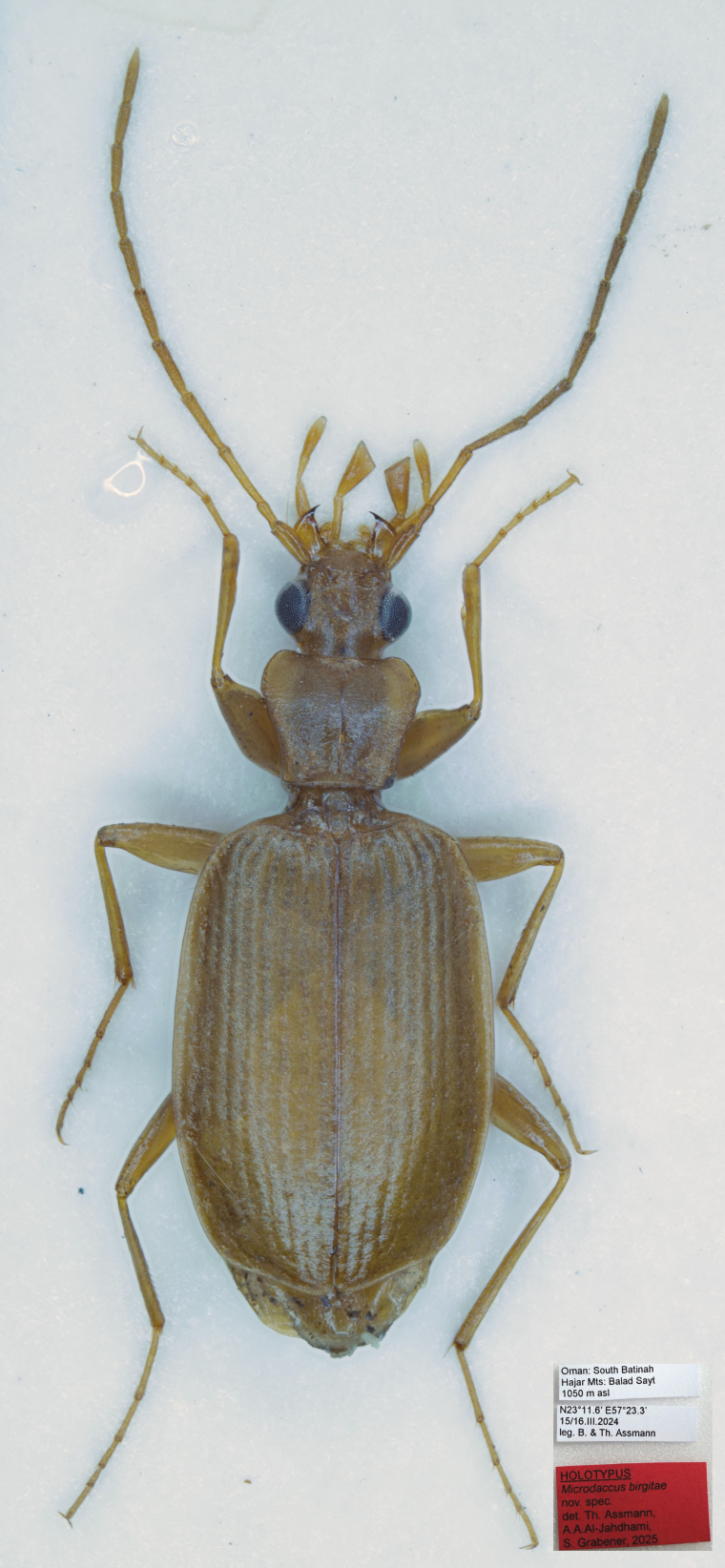
Habitus of *Microdaccus birgitae* sp. nov. in dorsal view, holotype, female, CAA; with labels.

**Diagnosis**. A large, light brown, fully winged *Microdaccus* species with slender head and pronotum, a small basal terebral tooth on each mandible, convex elytra with sinuously curved apex and long appendices (Fig. 1).

**Measurements**. Holotype: TL = 5.15 mm, HW = 0.88 mm, HW-E = 0.47 mm, AL = 3.60, PW = 1.06 mm, PB = 0.74 mm, PL = 0.85 mm, PC = 0.76, EW = 2.12 mm, EL = 3.10 mm.

**Colouration**. Light brown, diffuse darkened in the apical half of elytra, along the suture and the adjacent intervals, tip of mandibles black.

**Microsculpture**. The entire upper side with a strong isodiametric microsculpture, which makes the animal appear opaque. The lower side is shinier, but also predominantly with isodiametric, on the abdominal sternites with transverse microsculpture.

**Figure 3.**
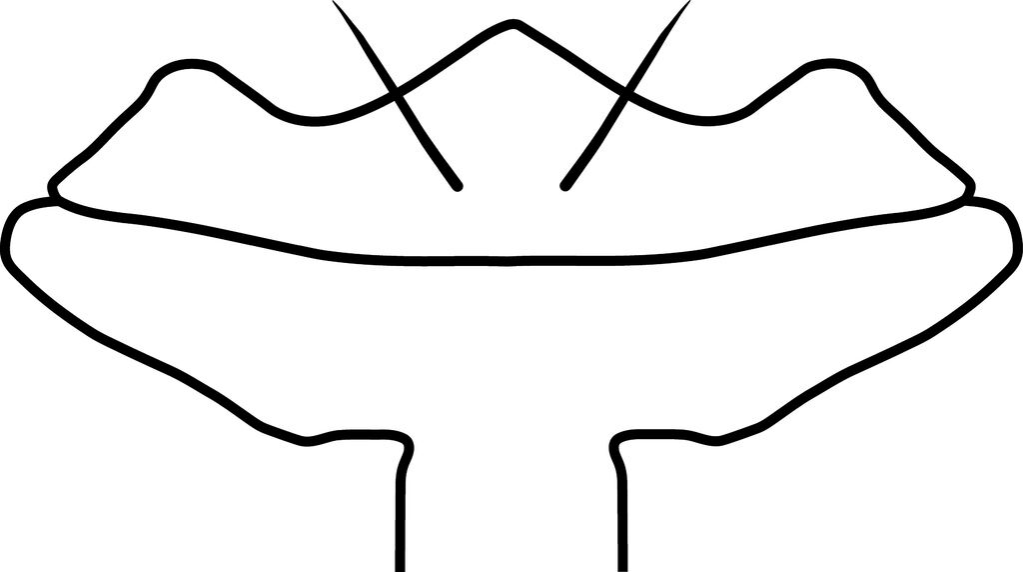
Mentum and submentum of *Microdaccus birgitae* sp. nov.

**Head** small, less than half width of elytra (HW/EW = 0.42, HW/PW = 0.83, PW/HW = 1.20), with protruding eyes (HW-E/HW = 0.59) covering about half of the width of the head; tempora short. Two pairs of supraorbital setiferous pores, the anterior seta close to the eyes mid-length and the posterior close to level of their posterior margin. From the anterior supraorbital setiferous pores to the antennal bases elevated, anteriorly diverging carinas; antennae inserted under the carinas. Frons subconvex, behind the anterior margin two lateral impressions prolongated to a V-shaped impression joining medially between the posterior margin of the eyes. Clypeus with two large setiferous pores, anterior margin straight, anterior angles sharp; posterior margin (= frontoclypeal suture) sinuate as posterior angles excised. Labrum with two clearly visible pairs and a small lateral pair of setiferous pores; anterior margin medially emarginated, rounded laterally. Mandibles strong, short and apically downwards curved, apex thin and pointed; with a small basal retinacular tooth, slightly below the incisior edge (terebra) from which the tooth is separated by an incision (Fig. 2a). Last segment of labial palpi triangular, about half as wide as long, longer than penultimate one. Maxillary palpi slender, last palpomere long, almost as wide as the third-last one, penultimate palpomere shorter than the last one and with one seta on inner side. Mentum with broad, only slightly rounded tooth that protrudes clearly beyond the lateral rounded lobes (Fig. 3). Antennae long, about 1.13 times as long as EL, 3^rd^ segment longer than 4^th^ one and almost 3 times longer than 2^nd^ one; from 4^th^ antennomere onwards with fine hairs (and sensillae), 1^st^ antennomere with a long distal seta, the other antennomeres with small distal setae.

**Pronotum** small (PW/EW = 0.50, PL/EL = 0.27), slender (PW/PL = 1.24, PW/EL = 0.34); cordiform; anterior angles rounded slightly protruding, anterior margin slightly concave; lateral gutter in the apical third widest, narrowest near the marginal setae, lateral sides in basal third almost parallel-sided, almost not widened to hind angles (PW/PB = 1.43, PW/HW = 1.20, PW/EW = 0.50, PC/PB = 1.03); median sulcus deep; basal impression deep and broadly connected to lateral gutter; pronotal lateral margin strongly upwards bent, proepipleura visible from above (Fig. 4a).

**Elytra** large, oval, (EL/TL = 0.60, EL/PL = 3.65, EL/EW = 1.46), broadly rounded, widest in the middle and the apical third, humeri rounded, lateral margins evenly rounded, apical margin sinuose (Fig. 5a). Disc slightly flattened. Scutellum small. Basal margin sinuate; scutellar setiferous pore at the beginning of 2^nd^ stria; intervals slightly convex, striae moderately deep and slightly punctate; inner striae and intervals relatively complete, but both outer striae and intervals flattened at apex, with a small pore at the end of the the 3^rd^ interval (no seta visible). Series umbilicata with 15 setiferous pores (arranged in groups as follows: 6 – 1 – 2 – 2 – 4), three pre-apical setiferous pores at end of 1^st^, 2^nd^ and 8^th^ striae (Fig. 5b) (the interval between 7^th^ and 8^th^ striae is bent inwards at the apex and thus shortens the previous intervals).

**Legs** long and slender; protibia on upper side with grooves, longer than protarsi; meso- and metatibiae slightly longer than respective tarsi. Tarsi including onychium on upper side pubescent, onychium on the lower side with two longitudinal rows of fine hairs; claws long, their inner side smooth.

**Figure 6.**
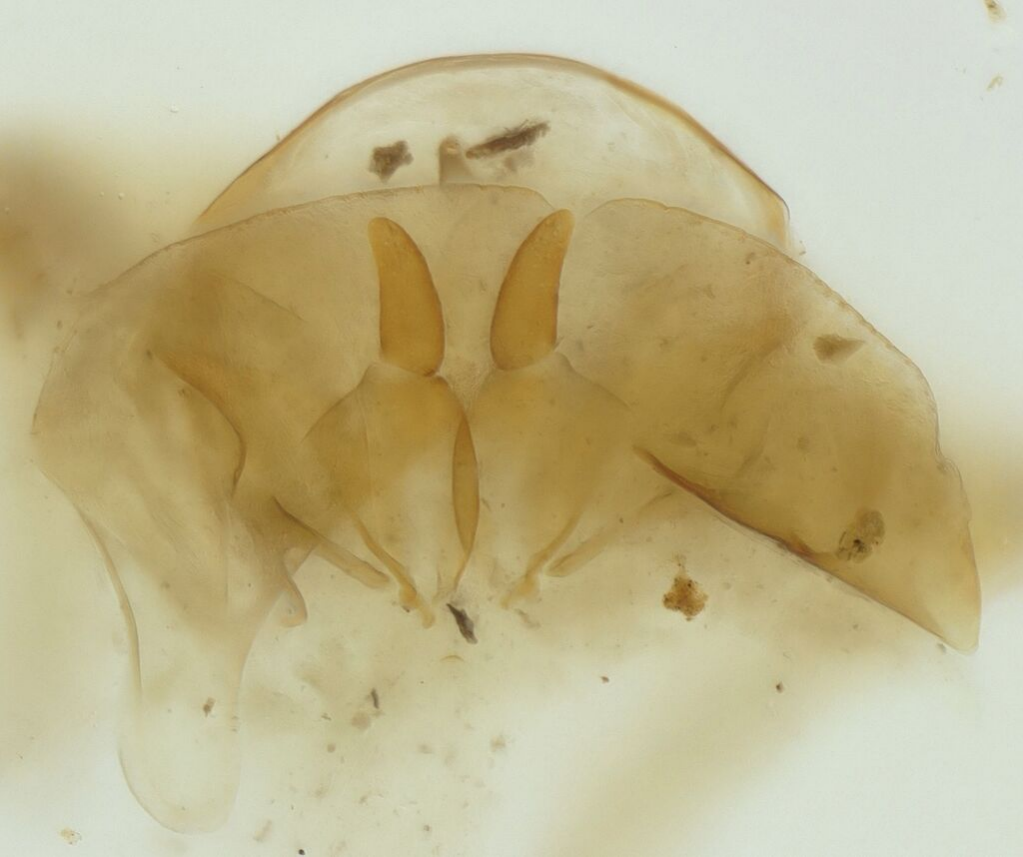
Gonocoxites of *Microdaccus birgitae* sp. nov.

**Female genital (IX^th^) segment** well developed; gonocoxite 1 about 1.5 longer than wide, gonocoxite 2 slightly bent and about 2.5 times longer than wide (Fig. 6).

**Comparison**. The slender pronotum with its almost straight lateral sides in the basal third is a unique character within the genus. The evenly rounded elytra with rounded humeri occur within the genus *Microdaccus* in only one other species, namely *M. bushehricus* Muilwijk & Kabak, 2019 from Iran (Muilwijk et al. 2019). *Microdaccus birgitae* sp. nov. is similar to this species, especially as it is also light brown in colour, has triangular ultimate labial palpomere etc. Both species bear fine hairs on the lower side of the onychium (Kabak personal communication). However, the new species differs from *M. bushehricus* in less markedly bent mandibles, three pre-apical setae on the elytra, sinuose apical elytral margin and in the shape of the pronotum. Lateral sides of the latter are almost rectilinear in basal half (deeply sinuate in *M. bushehricus*).

#### Etymology

This species is dedicated to Birgit Assmann, an enthusiastic amateur biologist and conservationist, as well as the wife of the first author. The first author discovered the new species together with her.

#### Distribution

The type specimen was found in the western Hajar Mountains on the north side of the ridge at an altitude of approximately 1,000 m above sea level. Several days of searching in December 2024 and March 2025 near the typical location close to the village of Balad Sayt were unsuccessful (incl. flight interception trap with UV-light).

#### Ecology

In March 2024, the beetle was active after sunset and extremely agile (ambient temperature approx. 24°C).

**Figure 7.**
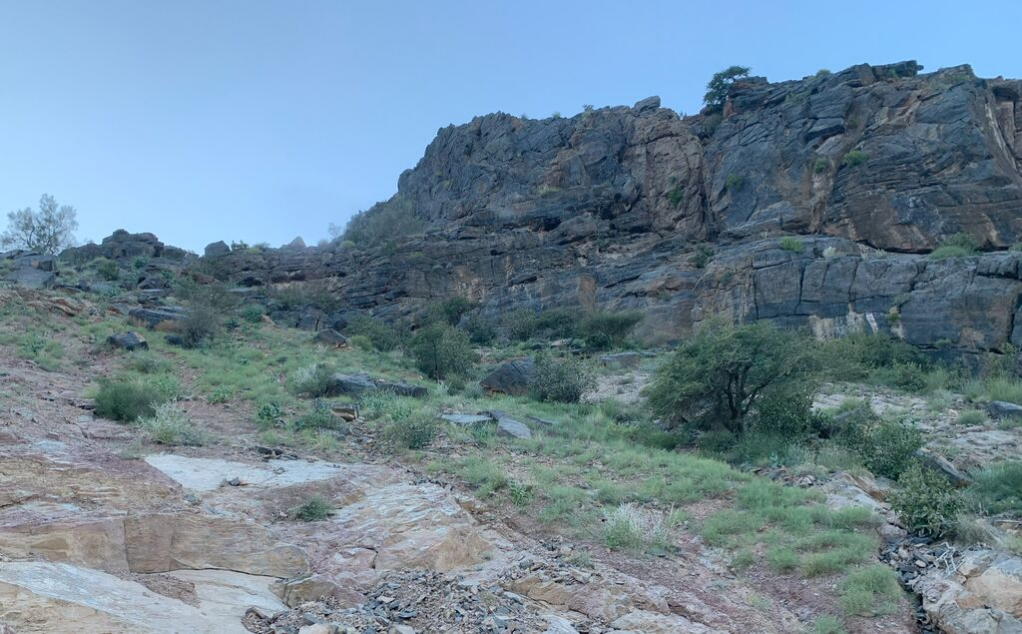
Habitat of *Microdaccus birgitae* sp. nov.

The vegetation of this arid to semi-arid submontane region consists of a mixture of open xeromorphic woodlands and semi-evergreen shrubland and belongs to the *Euphorbia larica*-*Moringa peregrina* community (Patzelt 2015), (Fig. 7).

### Microdaccus

Schaum, 1864

B591DE57-9C94-5CAC-BD90-BFC3C41CDDD9

#### Notes

*Sota and Kubota (1998)Microdaccus* and *Psammodromius* have been classified by most authors in recent decades as closely-related taxa belonging to Dromiusina Bonelli, 1810 (Morvan 1977, Mateu 1981, Lorenz 2005, Kabak 2017a). The subtribe is distributed globally and it comprises numerous genera (Lorenz 2005). However, as discussed by Rasool et al. (2018), there are differences amongst some authors regarding which genera belong to this subtribe. Baehr and Will (2019) provide probably the best characterisation of the subtribe. However, it is unclear whether this characterisation also applies to the generic taxa in Europe and Asia, but it covers the morphology of *Microdaccus* and *Psammodromius*.

Considering the difficulties in delineating the subtribe, it is not surprising that there are no clear delineations of the numerous genera in Europe and southern Asia. However, the generic taxa *Microdaccus* and *Psammodromius* have been discussed by Morvan (1977) and Mateu (1981) and the latter author characterised both generic taxa. Deviations from Mateu's description show the two species *M. birgitae* sp. nov. and *M. bushehricus*: the sides of the elytra are not almost parallel, but broadly rounded, including the humeri. Setal pores in the third elytral interval occur in the latter species (and in *M. glasunovi*). The onychium of *M. birgitae* sp. nov. also has very fine small hairs on the lower side (see above; Muilwijk et al. (2019)).

Rasool et al. (2018) have characterised the Dromiusina genera of the southern Arabian Peninsula by a combination of character states. Following their discrimination scheme, the genus *Microdaccus* can be easily recognised by a unique combination of: (a) its short antennomere II in comparison to antennomere III; (b) pubescence from antennomere IV and (c) the long hind tarsomere I in comparison to hind tarsomere V. The character states (b) and (c) are not so evident in *M. glasunovi* (Kabak personal communication).

### Psammodromius

Peyerimhoff, 1927

40C29D14-4CA5-550D-A8CF-9BE8E17304E7

#### Notes

*Psammodromius* was described as a genus by Peyerimhoff (1927). He compared the genus to *Dromius* Bonelli, 1810 and was probably unaware of the genus *Microdaccus*. Morvan (1977) and Mateu (1981) recognised the relationship between the two taxa and critically discussed possible differential characteristics. In addition, both authors conclude that only the genitalia differ consistently between the two taxa. In *Psammodromius*, the median lobe of the aedeagus has a long copulatory piece resembling a flagellum. A comparable form of a copulatory piece is not known from the genus *Microdaccus*.

Kabak (2017b) highlights the striking similarities between the taxa *glasunovi* Emetz, 1979 and *zajtzevi* (Eichler, 1924). He essentially only finds differences in the median lobe of the aedeagus and wonders whether these striking similarities do not rather argue in classifying Psammodromius as a subgenus. However, Kabak (2017b) called for further research.

Strong differences in the genitalia occur within numerous ground beetle genera (e.g. *Bembidion* Latreille, 1802, *Leistus* Frölich, 1799). Moreover, some genera that were established exclusively or predominantly on the basis of differences in the genitalia turn out to be paraphyletic (e.g. some tiger beetles, see Gough et al. (2018)). The strong differences in the genitalia of species belonging to other carabid genera, which even allow for interspecific gene flow (at least sometimes, for example, Sota and Kubota (1998), Sota and Vogler (2001), Deuve et al. (2012)), indicate that differences in the genitalia should not be solely used for the separation of genera in carabidology. Furthermore, no male genitalia are available for the three *Microdaccus* species described most recently (the holotypes of *M. bushehricus* and *M. sugonjaevi* are males, but the median lobes of the aedeagus are missing and the holotype of *M. birgitae* sp. nov. is a female). Formally, these three species cannot be assigned to either of the two genera. To avoid overemphasising the genitalia in the classification of genera, we recommend considering Psammodromius Peyerimhoff, 1927 stat. nov. as a subgenus of *Microdaccus* Schaum, 1864.

### Microdaccus (Microdaccus) opacus

(Schaum, 1857)

39048D60-A701-59C2-8636-80B4E2BC316A

#### Description

**Figure 8.**
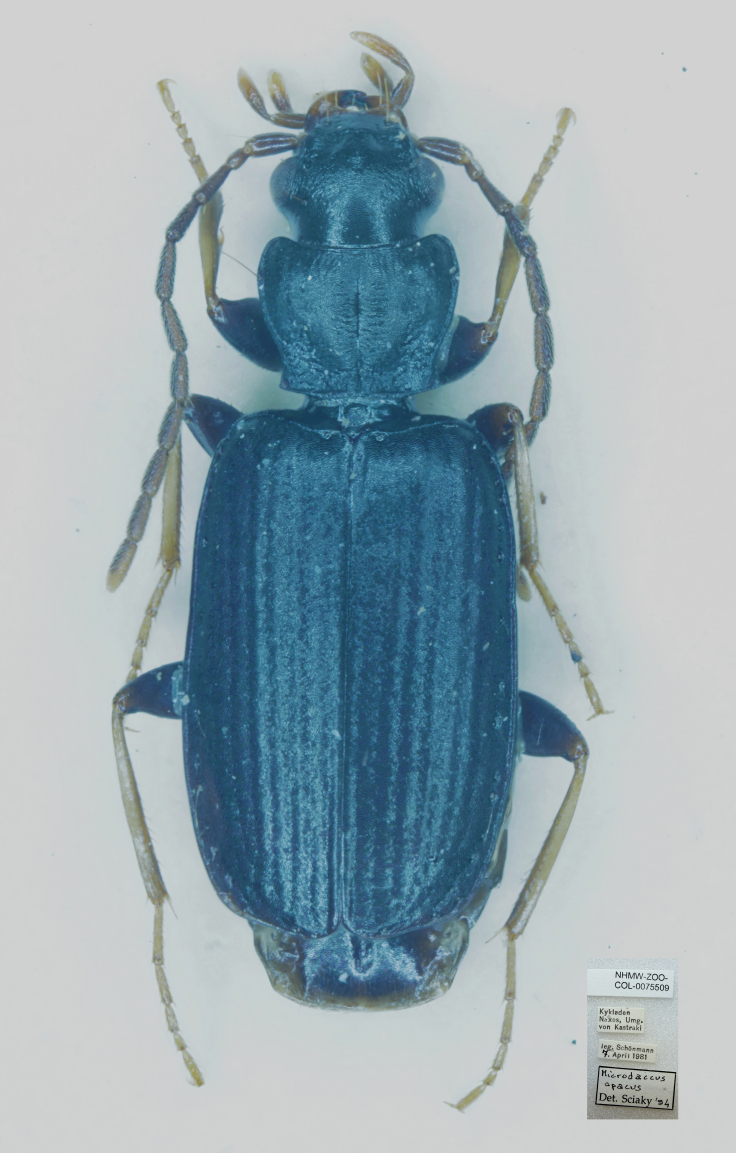
Habitus of *Microdaccus opacus*, female, NHMW; with labels.

The type species of *Microdaccus* is *M. opacus* (Schaum, 1857) which is particularly distinguished by its dark elytral colouration (Fig. 8) and mandibles with small teeth on their inner edge.

#### Distribution

Recorded from Greece and western Turkey (Kabak 2017a, Mateu 1981), but occurrence on Cyprus is unlikely; see *Microdaccus assingi*.

### Microdaccus (Microdaccus) assingi

Wrase, 2009

E0C37DAA-0487-564A-9409-79BA3DC6F93B

#### Notes

**Figure 9.**
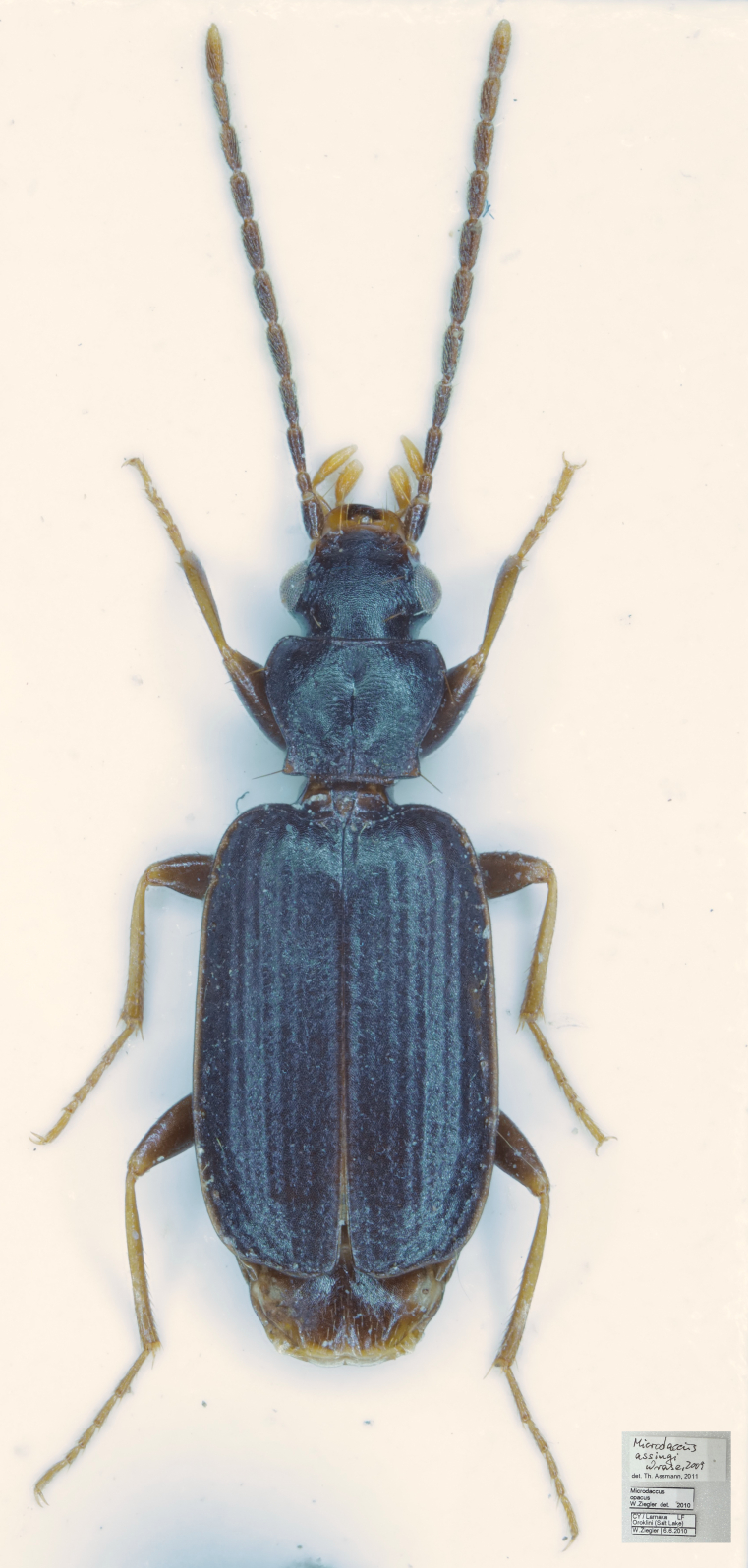
Habitus of *Microdaccus assingi*, female, CAA; with labels.

*Microdaccus assingi* was split off from *M. opacus* by Wrase (2009) (Fig. 9). In addition to the median lobe of aedeagus with small copulatory pieces (Fig. 10a), body length is a good distinguishing feature. The colouration of the antennae is more variable in both species than stated in the original description. We did not see *M. opacus* from Cyprus. In many collections, the small specimens in series that had been identified as *M. opacus* and originated from Cyprus were in fact *M. assingi*. It is very probable that only the latter species occurs on this island.

### Microdaccus (Microdaccus) pulchellus

Schaum, 1857

145F0C67-E924-5780-BFA5-6C39FF5CF5D3

#### Description

**Figure 11.**
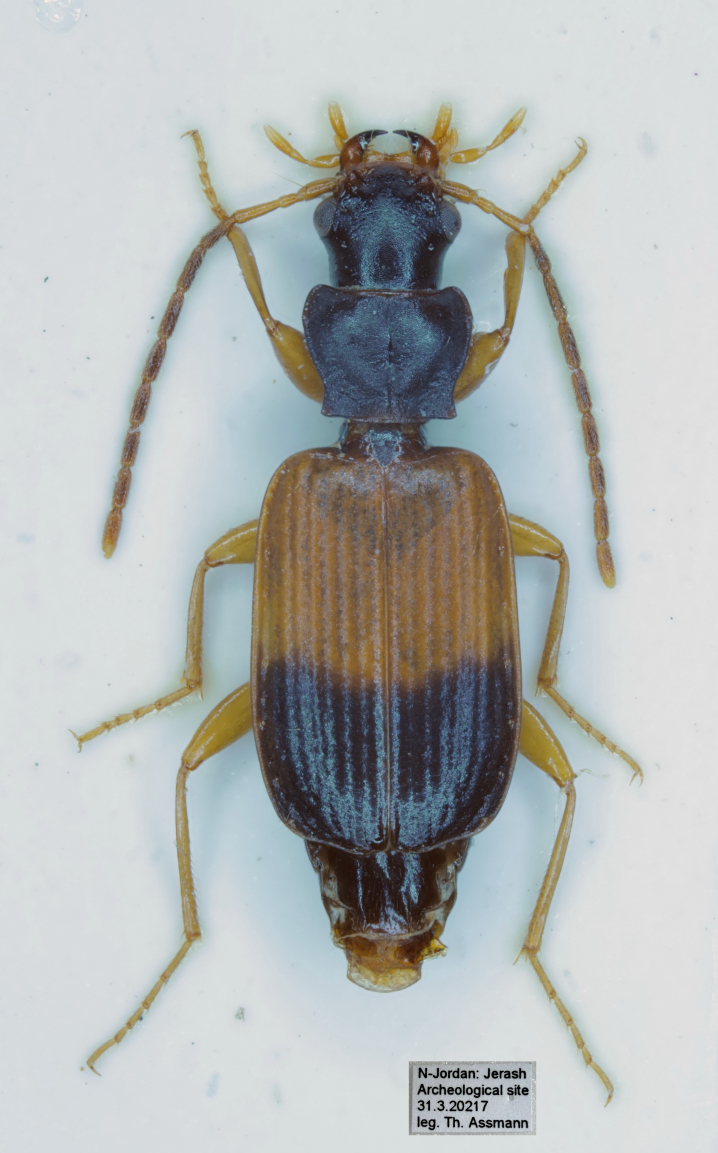
Habitus of *Microdaccus pulchellus*, female, CAA; with labels.

The species is characterised by its wide pronotum with less curved sides (Fig. 4b) and the bicolored elytra, the apical darkening of the elytra without any extension along the suture, femora often yellow-brownish as tibia and tarsus and a dark scutellum (Fig. 11).

#### Distribution

*Microdaccus pulchellus* was described by Schaum (1857) from Palestine and is only known to us from the southern Levant. We are not aware of any sympatric occurrence of *M. opacicolor* (see below) and *M. pulchellus*. Chikatunov et al. (2006) and Kabak (2017a) cite both species from Israel, but the collection in SMNH only contains *M. pulchellus*. We are only aware of the latter taxon from Lebanon, Israel and Jordan, but not from Turkey. However, two specimens from Turkey exist in the collection of Walter Heinz (Stuttgart) (Muilwijk, pers. com.).

### Microdaccus (Microdaccus) opacicolor

(Reitter, 1897)

CC46CAC1-0CB5-57A1-886E-C7FA9CD9B6F5

#### Distribution

We know this taxon from Turkey to Iran, but not from Israel (see *M. pulchellus*) and Lebanon.

#### Notes

*Microdaccus opacicolor* was described by Reitter (1897) as a member of the genus *Dromius* Bonelli, 1810 and later transferred to *Microdaccus* (Reitter 1900). When Reitter described *M. opacicolor*, he was probably unaware of *M. pulchellus*, but in 1900, he compared the two species and highlighted differences in the colouration of femora and the body lengths.

The type locality is Akbés in northern Syria (probably the Kurdish village of Meydan Ekbaz, which today belongs to Turkey). We found the holotype in the Frey Collection (NMBS) (Fig. 12), but the specimen is not well preserved. For further studies, more material from type locality is necessary. Approximately 500 km eastwards, Schönmann and Schillhammer (NHMW) collected two specimens, characterised by strongly curved sides of the pronotum and a narrow pronotal base (Fig. 13). These animals correspond quite well to the original description, but the hind angles are acute: “Pronotum slightly wider than long, strongly heart-shaped, narrowing sharply towards the rear edge and strongly curved outwards in front of the sharply rectangular hind angles, sides very narrow and curved upwards, [...]” (Reitter 1897: 30; translated by the authors).

**Figure 14.**
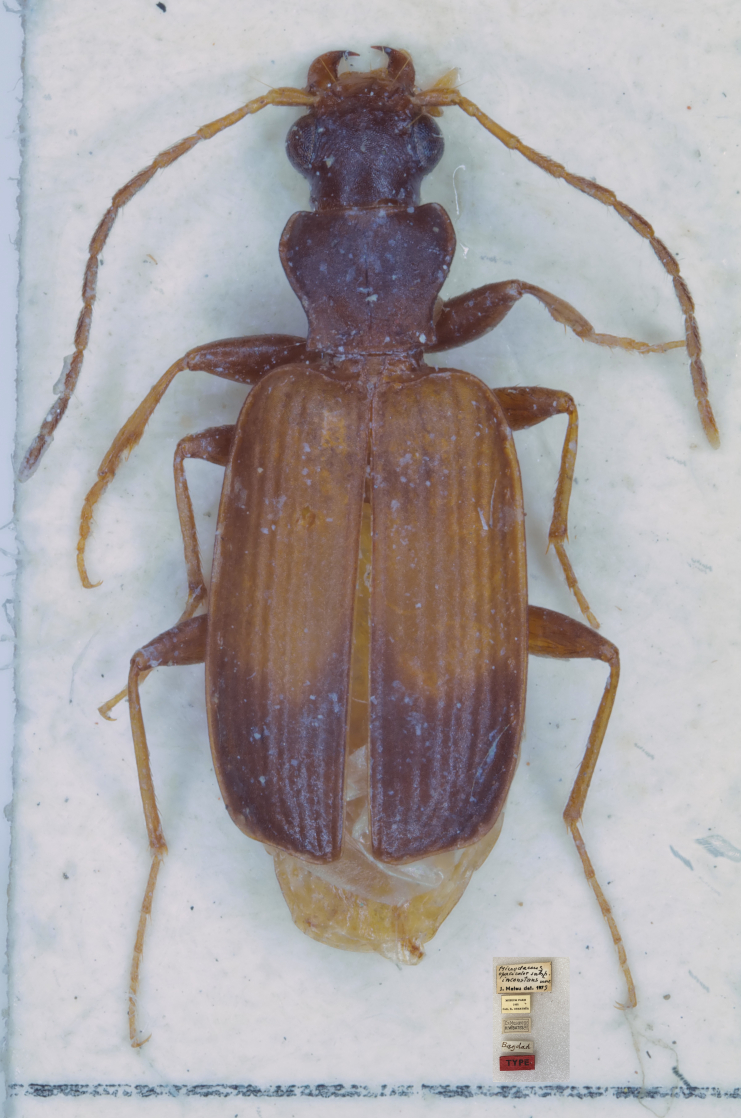
Habitus of *Microdaccus opacicolor inconstans*, holotype, male, MHMN; with labels.

**Figure 15.**
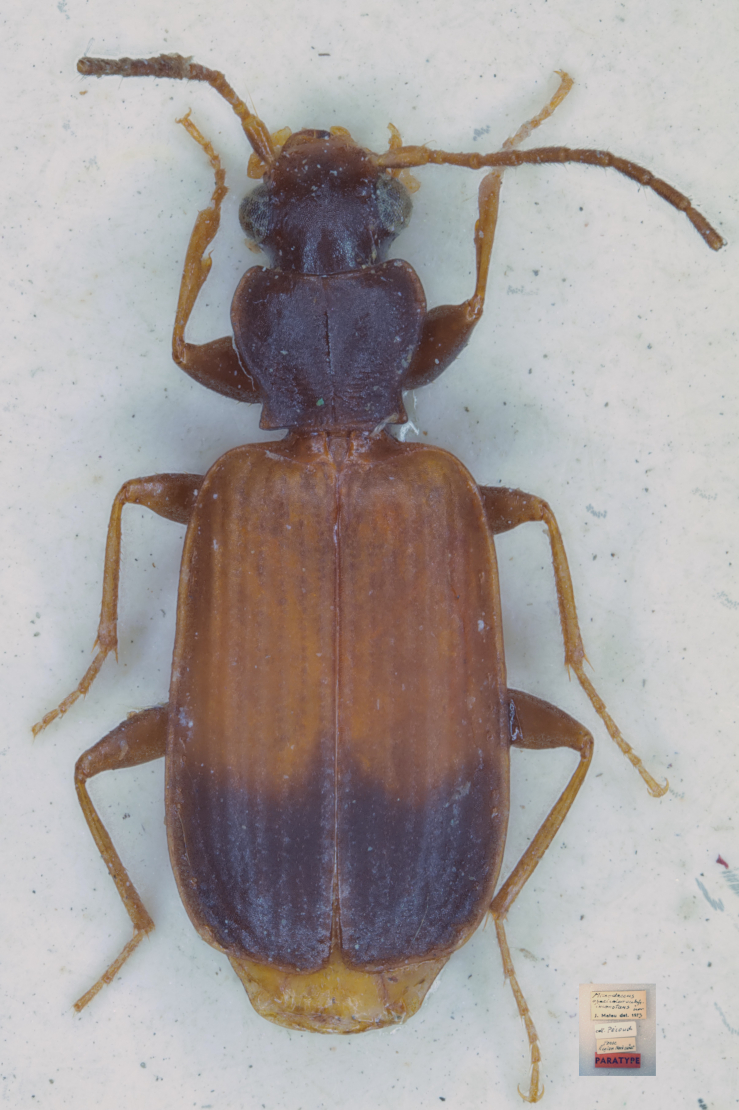
Habitus of *Microdaccus opacicolor inconstans*, paratype, female, MHMN; with labels.

**Figure 16.**
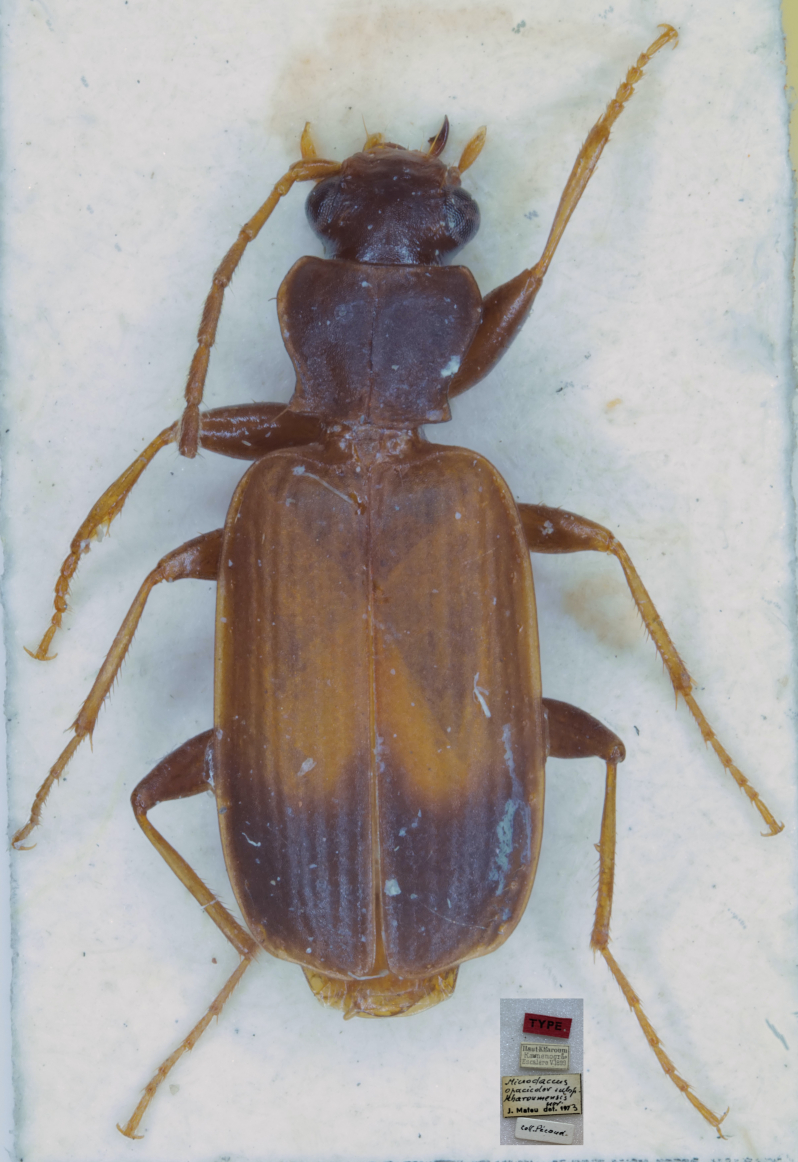
Habitus of *Microdaccus opacicolor kharoumensis*, holotype, male, MHMN; with labels.

The holotypes of the subspecies *M. opacicolor inconstans* Mateu, 1981 and *M. opacicolor kharoumanus* Mateu, 1981 differ from these specimens (Figs 14, 15, 16). However, at least some of the paratypes show clear infra-subspecific differences in the shape of pronotum and size ratios between elytra and pronotum. Mateu (1981) also included a female that is uniformly light brown in the paratype series of *M. opacicolor kharoumanus*. Whether this specimen really belongs to *M. opacicolor* remains unclear.

Mateu (1981) described the taxon *dubia* after a male from Turkey without any further indication of the locality. We examined the median lobe of the aedeagus embedded in a medium that has severely bleached the organ. As the median lobes of aedeagi are very similar or even identical in some specimens classified as *M. opacicolor* and *M. pulchellus*, we do not know distinguishing characters of *dubia* and the other subspecies of *M. opacicolor*.

We were unable to find any specimens of *M. escalerai* in the Morvan collection and can, therefore, only refer to Kabak’s classifications (Kabak 2017a).

**Figure 17.**
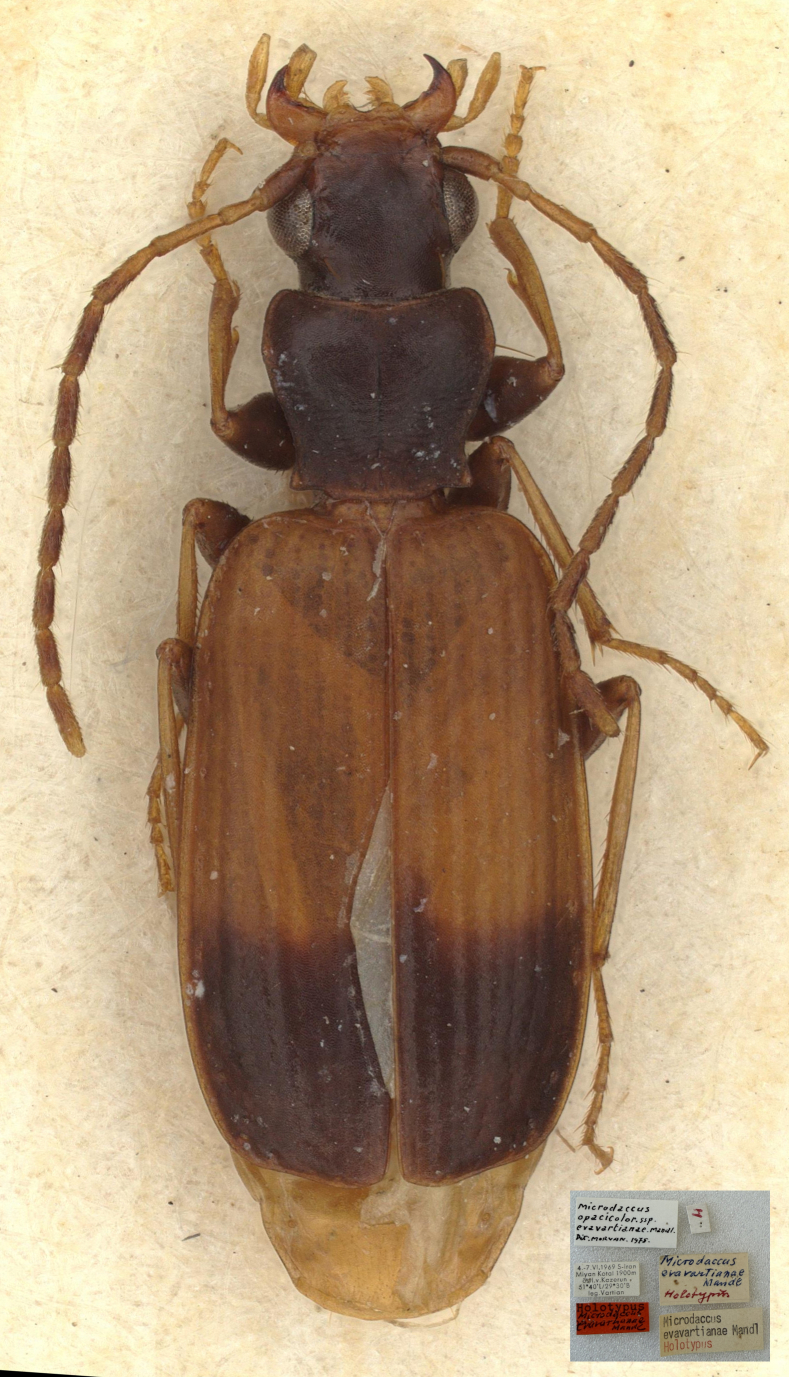
Habitus of *Microdaccus opacicolor evavartianae*, holotype, male, NMBS; with labels.

Mandl (1973) described the taxon *evavartianae* after a male from Iran (contrary to the description, which refers to a female, this specimen is actually a male; Muilwijk, personal communication). We were able to examine and photograph the holotype at the NMBS (Fig. 17). It falls within the range of variation of *M. opacicolor* and we have no reason to argue against the proposed classification of the taxon as a subspecies (Kabak 2017a); the same applies to the other taxa treated under *M. opacicolor*.

A final examination (and possible synonymisation) of the described subspecies can only be carried on the basis of additional material which allows the description of the intraspecific variability of all (or most) of the taxa, espcially from type locality of *opacicolor*. In the identification key, we rank *M. opacicolor* as a separate species and use the colouration and the shape of the pronotum as the main distinguishing feature from *M. pulchellus*.

### Microdaccus (Microdaccus) teodoroi

Gridelli, 1930

D9539163-289D-5750-92CF-253CCE784425

#### Description

**Figure 18.**
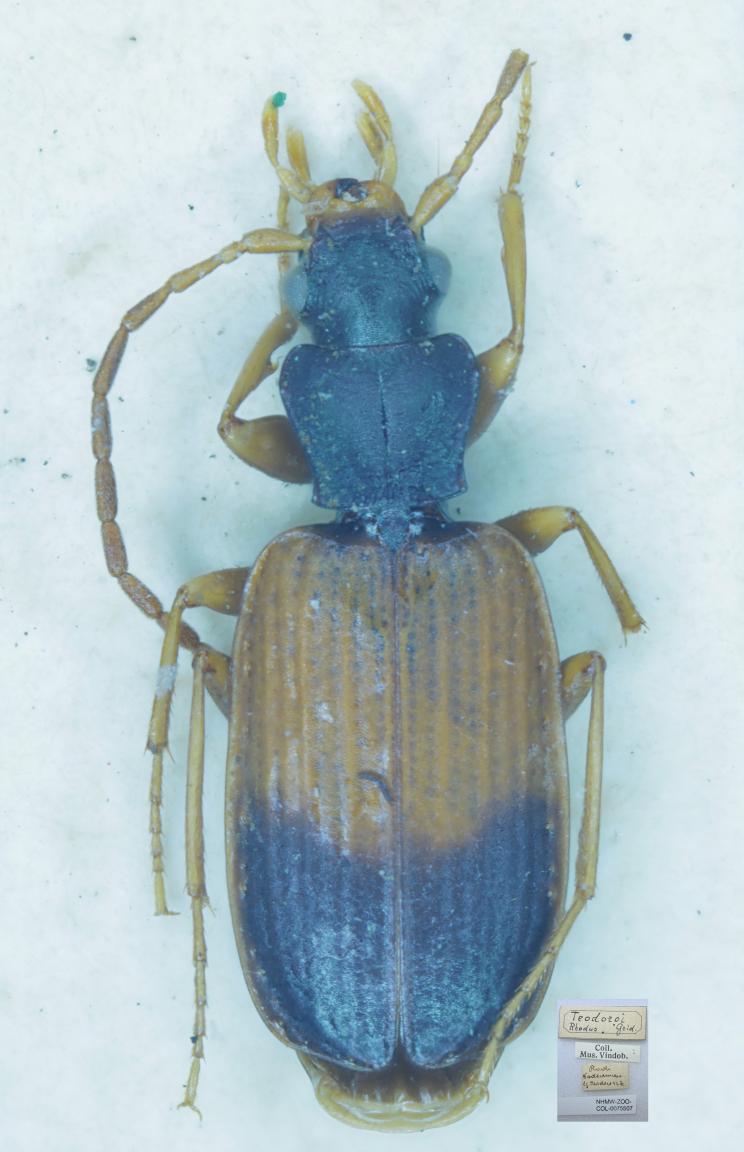
Habitus of *Microdaccus teodoroi*, male, NHMW; with labels.

This species has bicoloured elytra like *M. pulchellus*, but has a small tooth on the inside edge of both mandibles (Fig. 18).

#### Notes

Mateu (1981) states in his identification key that the species has elytra with an extension of the apical darkening along the suture and more strongly convex intervals. This does not correspond to Gridelli's description and our findings on three specimens in NHMW, one of them collected by Teodoro himself (Fig. 18).

### Microdaccus (Psammodromius) noctivagus

(Peyerimhoff, 1927)

F6B909B0-8393-5096-A0A4-3E91B942FD52

#### Description

**Figure 19.**
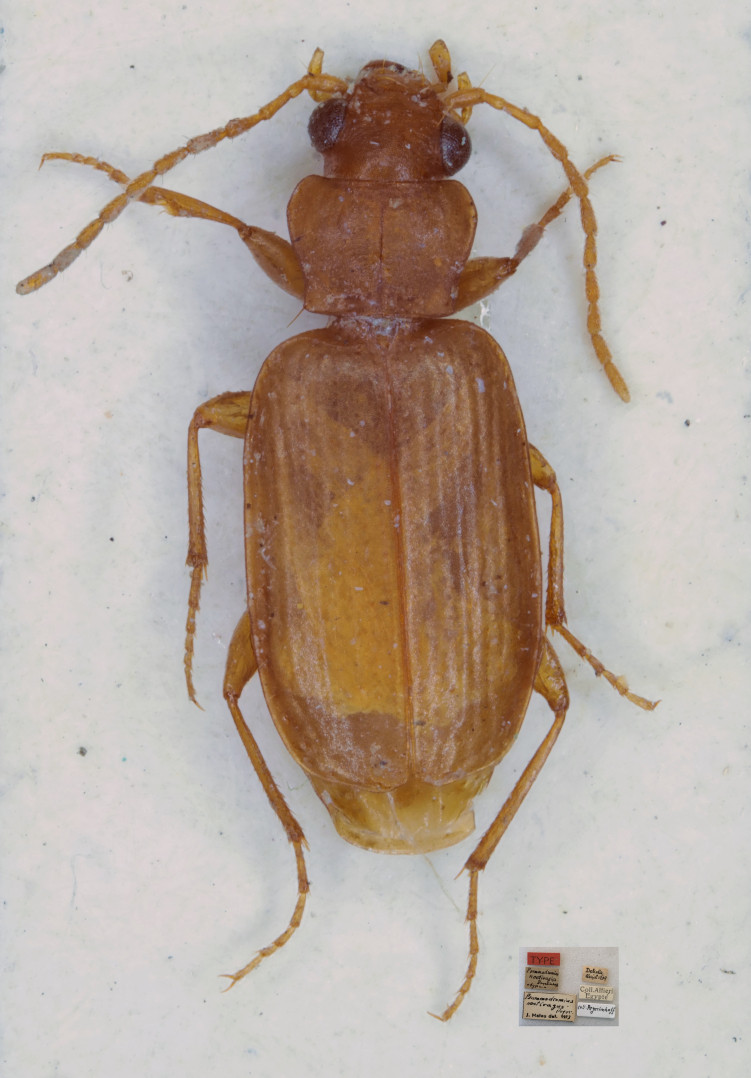
Habitus of Microdaccus (Psammodromius) noctivagus, holotype, female, MHMN; with labels.

**Figure 20.**
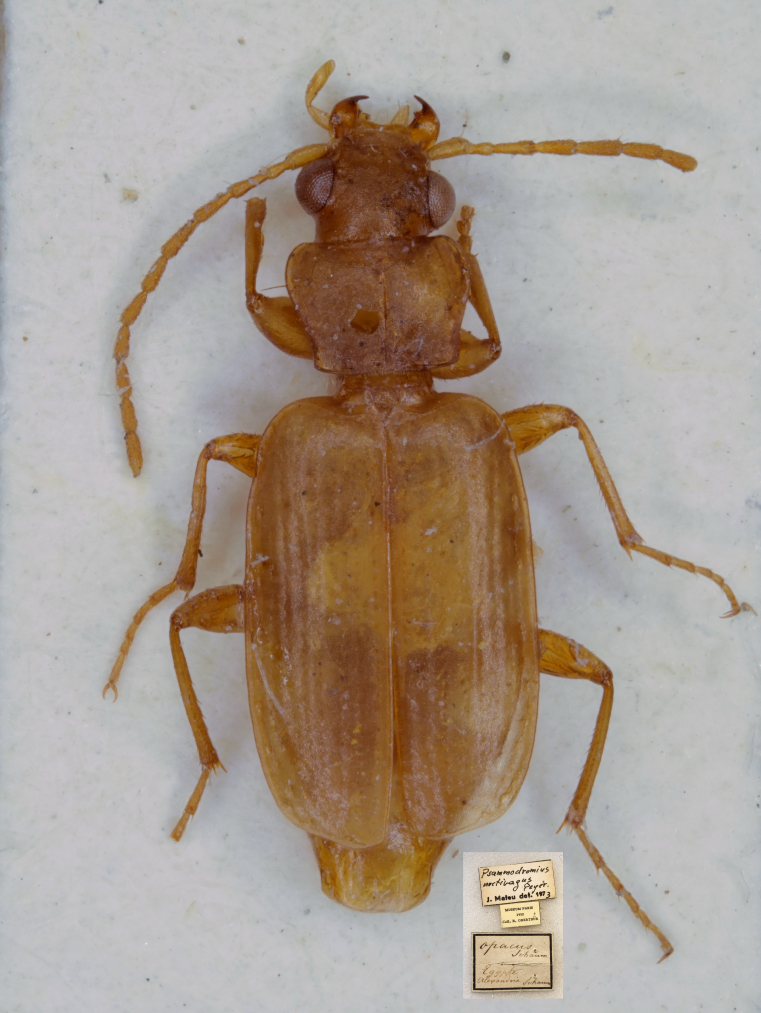
Habitus of Microdaccus (Psammodromius) noctivagus, male, MHMN; with labels.

The species differs from *M. zajtzevi* by the less cordiform pronotum (Figs 4c, 19, 20), larger body size and a median lobe of aedeagus which is enlarged in the apical half (Fig. 10d).

#### Distribution

*Microdaccus noctivagus* (Peyerimhoff, 1927) is a lesser-known species. We are not aware of any overlap with *M. zajtzevi*. The former species is known to occur in the Mediterranean coastal region of Egypt (Abdel-Dayem 2012, Alfieri 1976). We also have evidence of its occurrence in the Negev, east of Sede-Boqer (CAA).

### Microdaccus (Psammodromius) zajtzevi

(Eichler, 1924)

3F50B875-A03D-5203-B96F-54134A4B2DFB

#### Description

**Figure 21.**
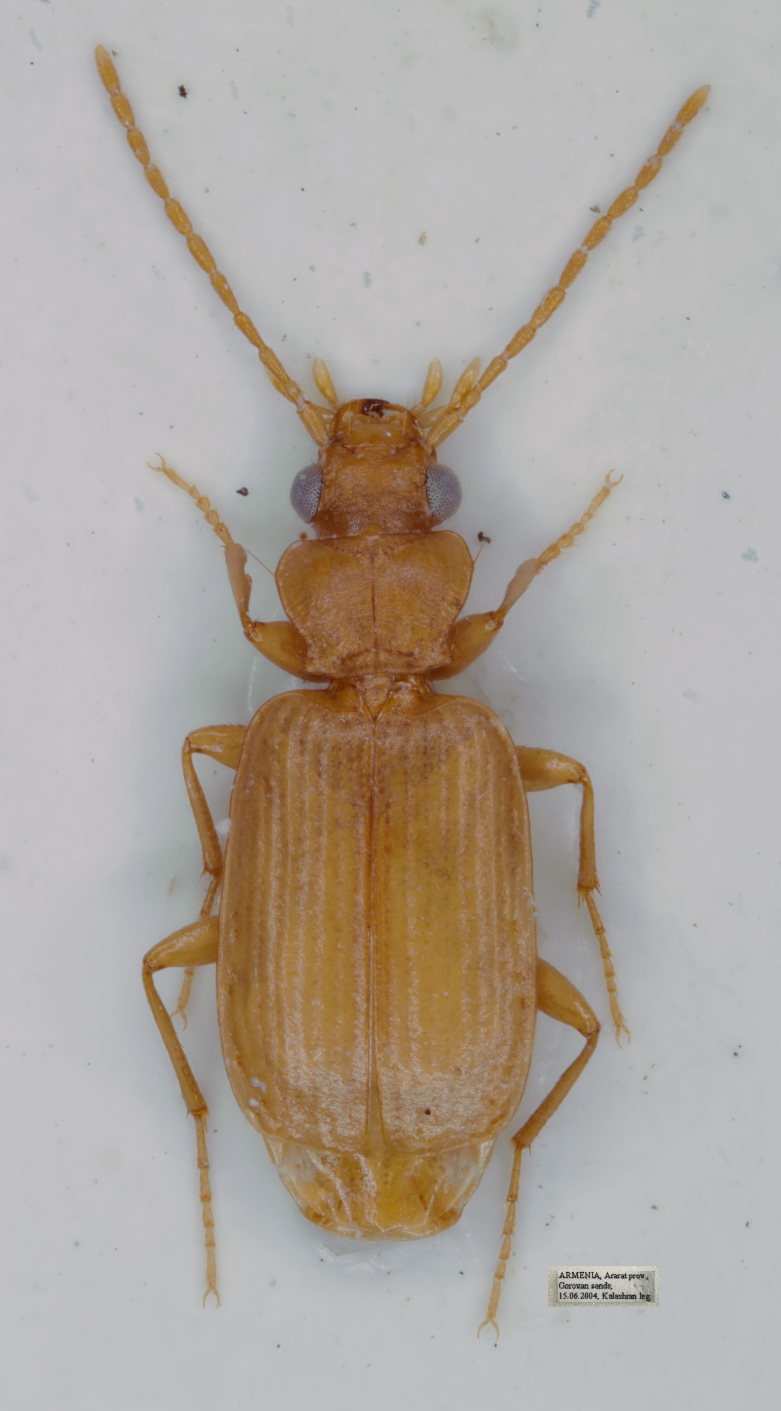
Habitus of Microdaccus (Psammodromius) zajtzevi, male, CAA; with labels.

The species differs from *M. noctivagus* by its smaller pronotum with more cordiform lateral sides (Fig. 21).

#### Distribution

*Microdaccus zajtzevi* occurs from Armenia and Turkey in the west to Turkmenistan and Iran in the east (Kabak 2017b). A distribution gap between this species and *M. noctivagus* is possible (see above).

#### Notes

For synonyms of *M. zajtzevi*, see Mateu (1981) and Kabak (2016).

## Identification Keys

### Identification key to the known *Microdaccus* species

**Table:** 

1	Both mandibles with one or two clearly visible teeth on the inner side (Fig. 2a, c).	2
–	Both mandibles without clearly visible teeth on the inner side of the mandibles (Fig. 2b).	5
2	Elytra homogeneously dark brown or black (Figs 8, 9).	3
–	Elytra not homogeneously dark brown or black.	4
3	Larger species, in general between 4.5-5.0 mm long. Median lobe of aedeagus with copulatory pieces longer than 1/5 of total length (Figs 8, 10b). Greece and western Turkey.	*** Microdaccus *** **(s.str.) opacus (Schaum, 1857)**
–	Smaller species, in general between 3.3-4.0 mm long. Median lobe of aedeagus with copulatory pieces less than 1/5 of total length (Figs 9, 10a).	**Microdaccus (s.str.) assingi Wrase, 2009**
4	Head and pronotum dark brown or black. Elytra bicolored: yellow-brownish or light reddish and large apical darkening, without any prolongation along the suture forwards (Fig. 18). 4-4.5 mm. Rhodos and Turkey.	**Microdaccus (s.str.) teodoroi Gridelli, 1930**
–	Head and pronotum yellow-brownish.	6
5	Scutellum dark brown or black. Apical darkening of the elytra without any extension along the suture. Femur often yellow-brownish as tibia and tarsus. Pronotum less cordiform (Fig. 11). 3.8-4.5 mm. Southern Levant: Lebanon, Israel, Jordan.	**Microdaccus (s.str.) pulchellus (Schaum, 1857)**
–	Scutellum yellow or brownish-red like the basal half of elytra. Apical darkening of the elytra with an extension along the suture. Femur often darker than tibia and/or tarsus (Figs 12, 13, 14, 15, 16, 17). 4.0-4.8 mm. Turkey to Iran.	**Microdaccus (s.str.) opacicolor (Reitter, 1897)**
6	Elytra and humeri strongly rounded. Legs long (Fig. 1).	7
–	Elytra parallel-sided or not continuously rounded, with protruding humeri.	8
7	Pronotum slender (Figs 1, 4a). 5.1 mm. Oman.	**Microdaccus (s.str.) birgitae n. sp**.
–	Pronotum more cordiform, the sides sinuous, see Fig. 1 in Muilwijk et al. (2019). 5.0 mm. Iran.	**Microdaccus (s.str.) bushehricus Muilwijk & Kabak, 2019**
8	Last segment of labial palpi apically enlarged, almost triangular (see Fig. 17 in Kabak (2015)). A small dark spot in the apical half of the elytra. 4.5 mm. Afghanistan.	**Microdaccus (s.str.) sugonjaevi Kabak, 2015**
–	Last segment normal or slightly enlarged, not triangular. Elytra homogeneously yellow or brown.	9
9	Copulatory pieces short, less than ¼ of total length of median lobe of aedeagus (see Fig. 3 in Kabak (2017b)). Pronotum strongly cordiform (see Figs 1, 2 in Kabak (2017b)) 3.9-4.3 mm. Kazakhstan.	*** Microdaccus *** **(s.str.)** ***glasunovi*** **Emetz, 1979**
–	Copulatory pieces much longer, about ½ of total length of median lobe of aedeagus, shape like a flagellum (Fig. 10d, e).	10
10	Larger, > 4.4 mm; median lobe of aedeagus at preputial field wider (Fig. 10d); microsculpture stronger; pronotum wider, less cordiform, hind angles less protruding, lateral sinuation of pronotal base shallow (*Figs 4c, 19, 20*). Egypt, Israel.	**Microdaccus (Psammodromius) noctivagus (Peyerimhoff, 1927)**
–	Smaller, < 4.2 mm, median lobe of aedeagus at preputial field slender (Fig. 10e); microsculpture weaker; pronotum smaller, more cordiform, lateral sinuation of pronotal base stronger (Figs 4d, 21). South of Caucasus, from Turkey to Iran and Turkmenistan.	**Microdaccus (Psammodromius) zajtzevi (Eichler, 1924)**

We did not see specimens of *M. bushehricus*, *M. sugonjaevi* and *M. glasunovi*. However, the detailed descriptions and illustrations given by Kabak (2015), Kabak (2017b) and Muilwijk et al. (2019) allow us to integrate the relevant species into our identification key. The type of *Microdaccus mirzayani* Morvan, 1977 was not available to us at MHMN. This taxon is not included in the identification key. Perhaps it is identical to *M. zajtzevi* (Kabak 2016).

## Supplementary Material

XML Treatment for Microdaccus (Microdaccus) birgitae

XML Treatment for Microdaccus

XML Treatment for Psammodromius

XML Treatment for Microdaccus (Microdaccus) opacus

XML Treatment for Microdaccus (Microdaccus) assingi

XML Treatment for Microdaccus (Microdaccus) pulchellus

XML Treatment for Microdaccus (Microdaccus) opacicolor

XML Treatment for Microdaccus (Microdaccus) teodoroi

XML Treatment for Microdaccus (Psammodromius) noctivagus

XML Treatment for Microdaccus (Psammodromius) zajtzevi

## Figures and Tables

**Figure 2a.**
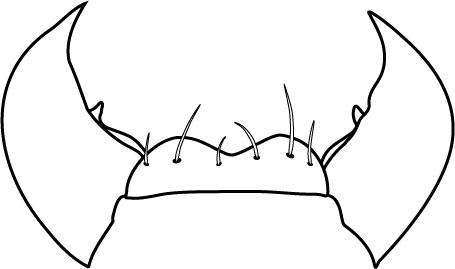
*Microdaccus birgitae* sp. nov.;

**Figure 2b.**
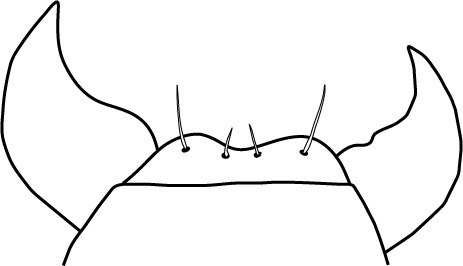
*Microdaccus pulchellus*;

**Figure 2c.**
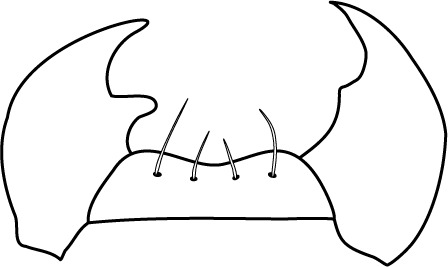
Microdaccus (Psammodromius) noctivagus.

**Figure 4a.**
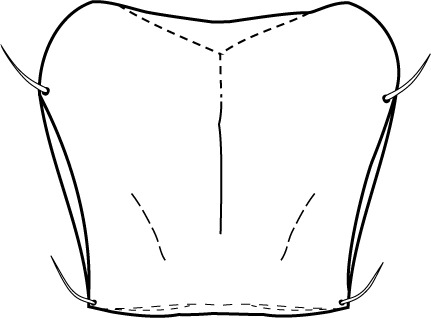
*Microdaccus birgitae* sp. nov.;

**Figure 4b.**
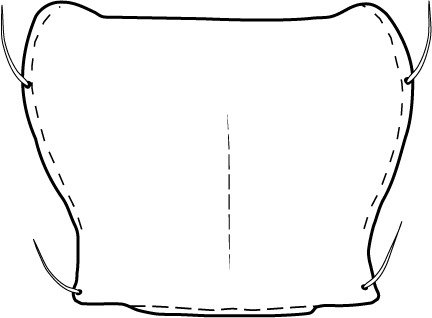
*Microdaccus pulchellus*;

**Figure 4c.**
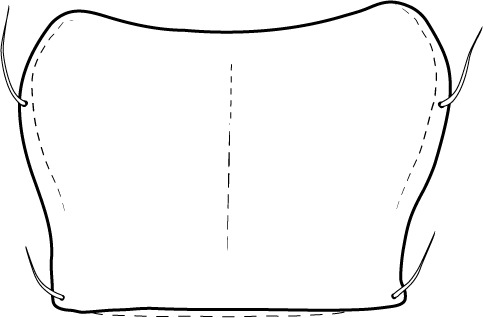
Microdaccus (Psammodromius) noctivagus;

**Figure 4d.**
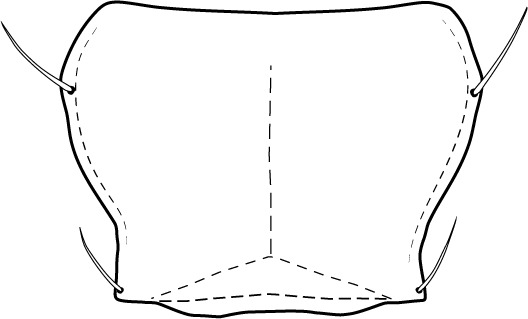
Microdaccus (Psammodromius) zajtzevi.

**Figure 5a.**
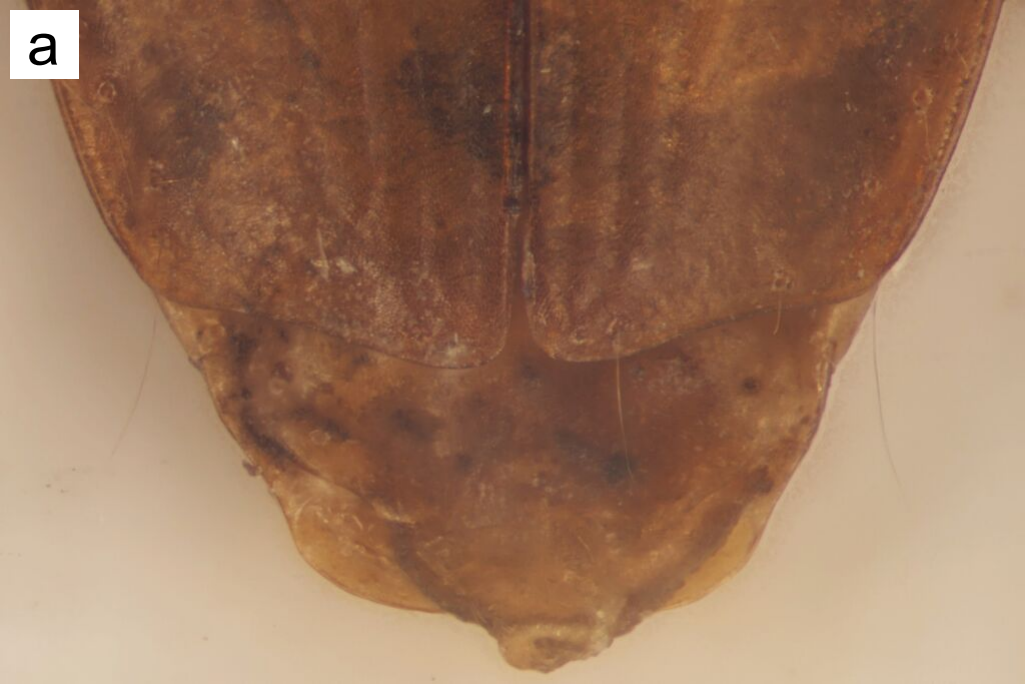
Elytral hind margin;

**Figure 5b.**
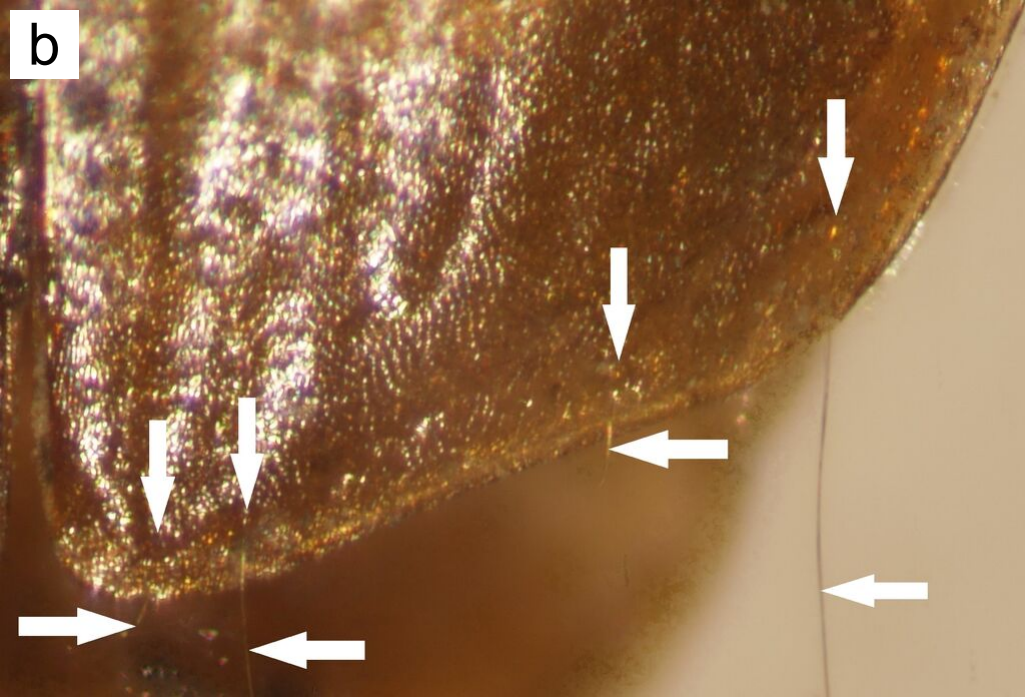
Apex of right elytron, horizontal arrows indicate setae, vertical arrows setal pores. The right pore and seta belong to the series umbilicata.

**Figure 10a.**
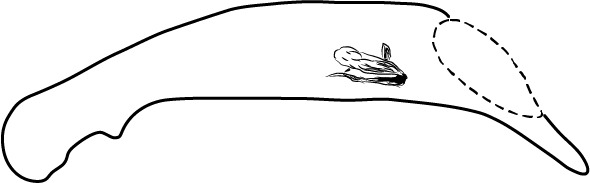
*Microdaccus assingi*;

**Figure 10b.**
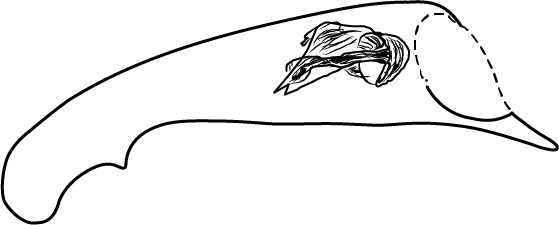
*Microdaccus opacus*;

**Figure 10c.**
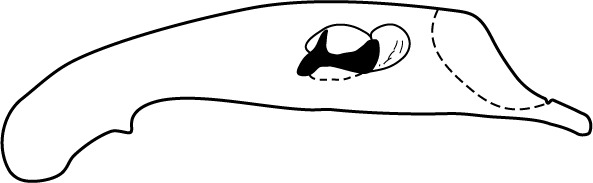
*Microdaccus pulchellus*;

**Figure 10d.**
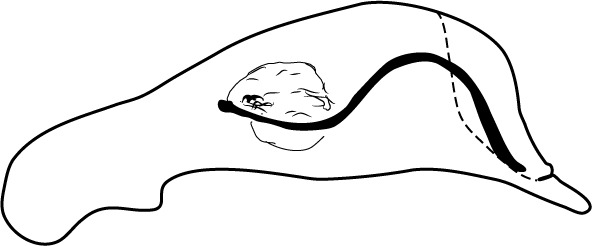
Microdaccus (Psammodromius) noctivagus;

**Figure 10e.**
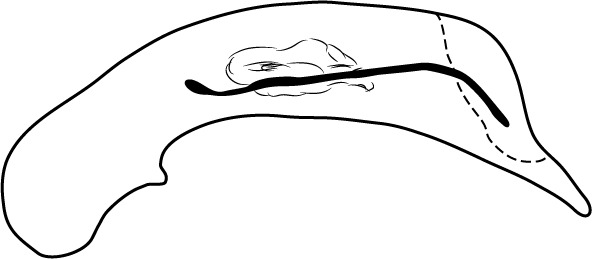
Microdaccus (Psammodromius) zajtzevi.

**Figure 12a.**
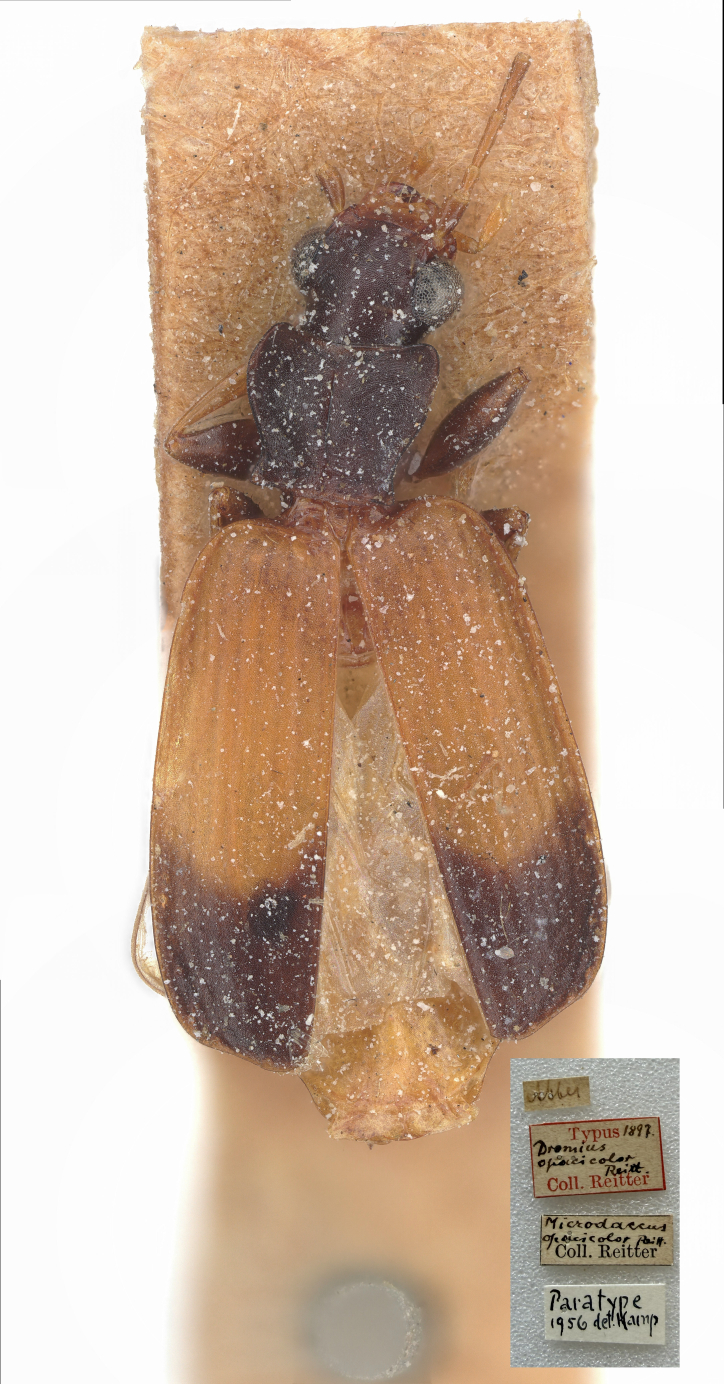
Habitus;

**Figure 12b.**
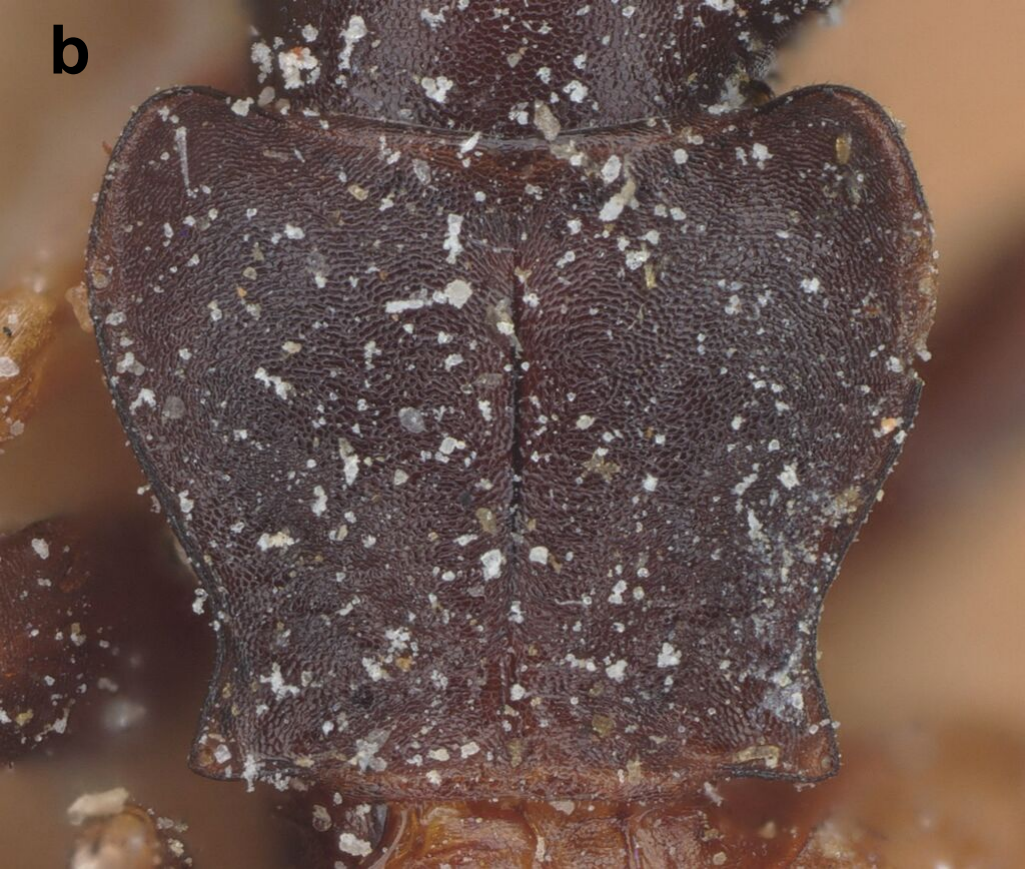
Pronotum in different viewing angle.

**Figure 13a.**
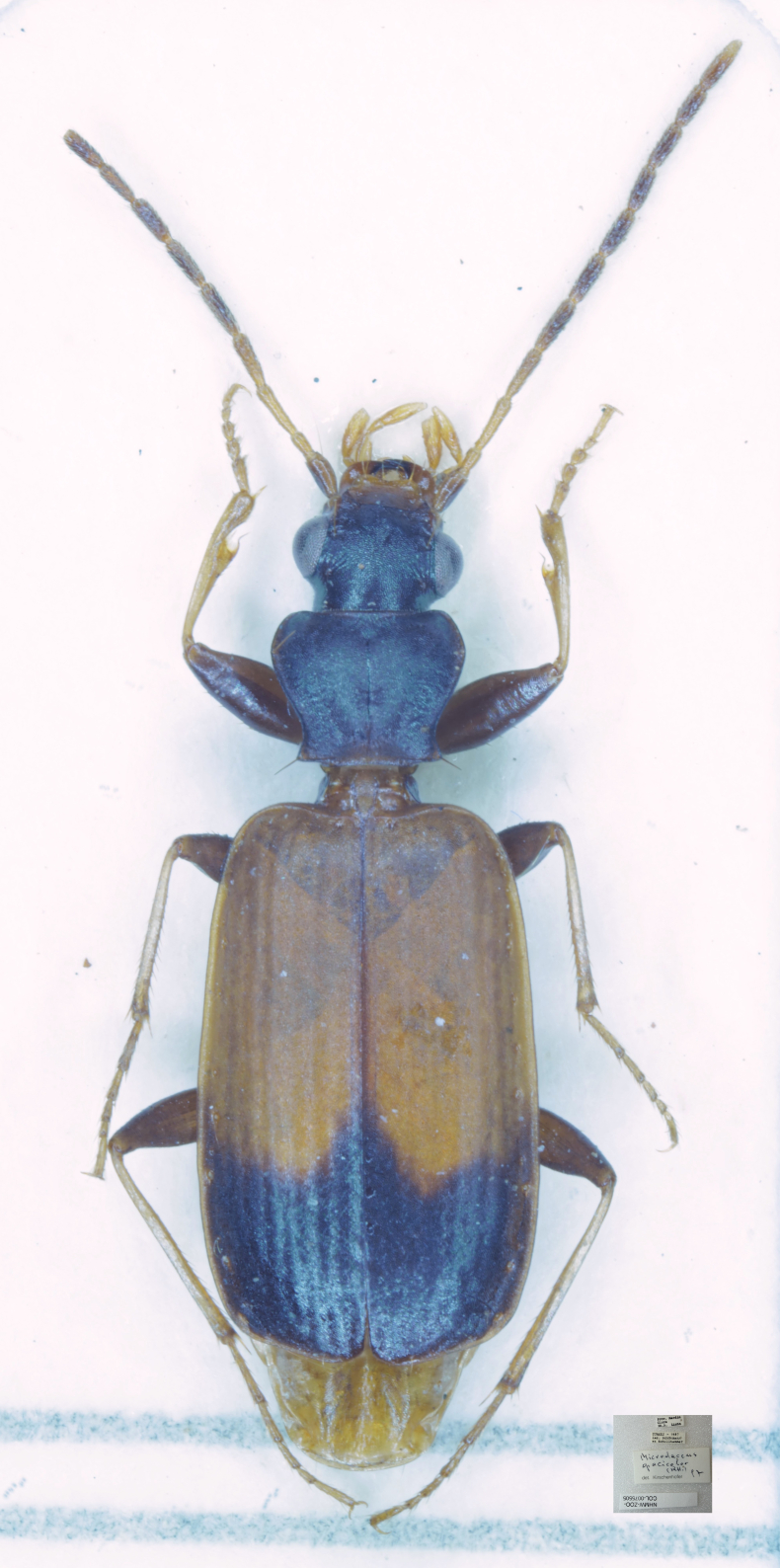
Habitus in dorsal view; with labels;

**Figure 13b.**
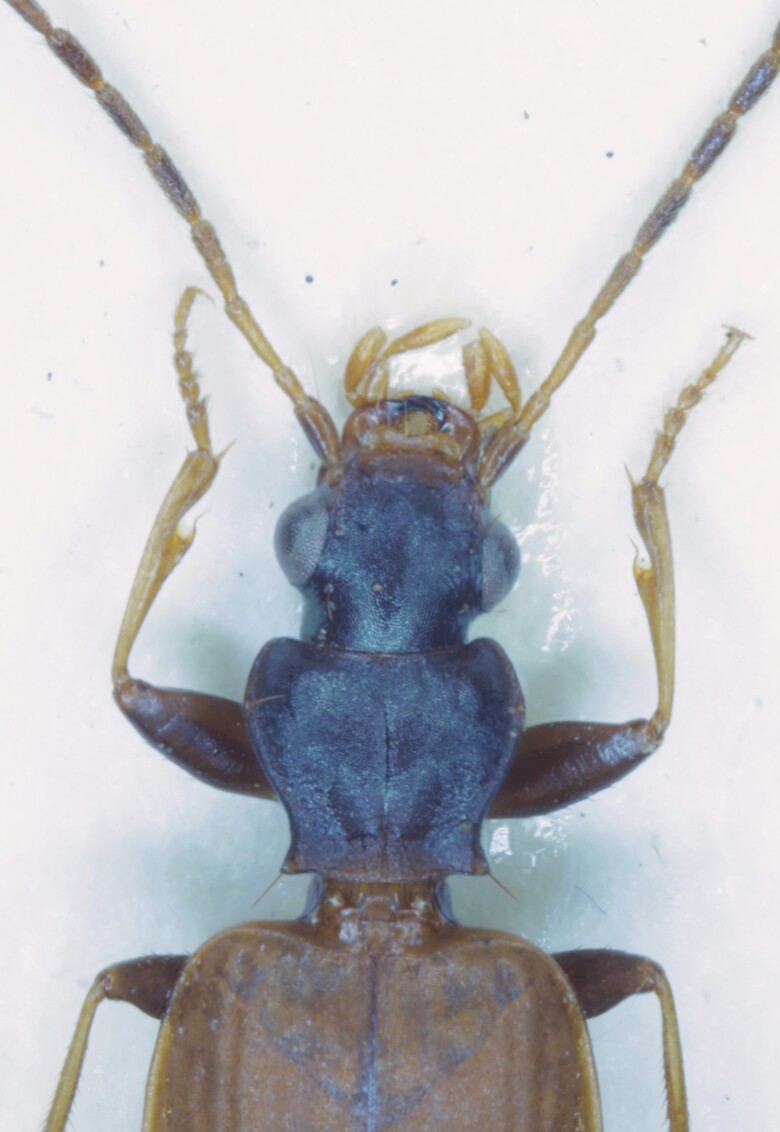
Fore-body in different viewing angle.
